# Physiologically Relevant Fluid-Induced Oscillatory Shear Stress Stimulation of Mesenchymal Stem Cells Enhances the Engineered Valve Matrix Phenotype

**DOI:** 10.3389/fcvm.2020.00069

**Published:** 2020-05-19

**Authors:** Brittany A. Gonzalez, Manuel Perez-Nevarez, Asad Mirza, Marcos Gonzalez Perez, Yih-Mei Lin, Chia-Pei Denise Hsu, Allen Caobi, Andrea Raymond, Mario E. Gomez Hernandez, Francisco Fernandez-Lima, Florence George, Sharan Ramaswamy

**Affiliations:** ^1^Cardiovascular Therapeutics Laboratory (CV-PEUTICS Lab), Department of Biomedical Engineering, Florida International University, Miami, FL, United States; ^2^Department of Immunology and Nano-Medicine, Florida International University, Miami, FL, United States; ^3^Advanced Mass Spectrometry Facility, Florida International University, Miami, FL, United States; ^4^Department of Chemistry and Biochemistry, Florida International University, Miami, FL, United States; ^5^Department of Mathematics and Statistics, Florida International University, Miami, FL, United States

**Keywords:** somatic growth, engineered valve, mechanical conditioning, physiologically relevant, mesenchymal stem cells, endothelial phenotype, smooth muscle phenotype, oscillatory flow

## Abstract

Support of somatic growth is a fundamental requirement of tissue-engineered valves. However, efforts thus far have been unable to maintain this support long term. A key event that will determine the valve's long-term success is the extent to which healthy host tissue remodeling can occur on the valve soon after implantation. The construct's phenotypic-status plays a critical role in accelerating tissue remodeling and engineered valve integration with the host via chemotaxis. In the current study, human bone-marrow-derived mesenchymal stem cells were utilized to seed synthetic, biodegradable scaffolds for a period of 8 days in rotisserie culture. Subsequently, cell-seeded scaffolds were exposed to physiologically relevant oscillatory shear stresses (overall mean, time-averaged shear stress, ~7.9 dynes/cm^2^; overall mean, oscillatory shear index, ~0.18) for an additional 2 weeks. The constructs were found to exhibit relatively augmented endothelial cell expression (CD31; compared to static controls) but concomitantly served to restrict the level of the activated smooth muscle phenotype (α-SMA) and also produced very low stem cell secretion levels of fibronectin (*p* < 0.05 compared to static and rotisserie controls). These findings suggest that fluid-induced oscillatory shear stresses alone are important in regulating a healthy valve phenotype of the engineered tissue matrix. Moreover, as solid stresses could lead to increased α-SMA levels, they should be excluded from conditioning during the culture process owing to their associated potential risks with pathological tissue remodeling. In conclusion, engineered valve tissues derived from mesenchymal stem cells revealed both a relatively robust valvular phenotype after exposure to physiologically relevant scales of oscillatory shear stress and may thereby serve to accelerate healthy valve tissue remodeling in the host post-implantation.

## Introduction

Heart valve disease requires a largely singular treatment in the form of artificial valve replacement. Indeed, prosthetic valve replacement devices offer reasonable solutions; however, durability and risk factors are of concern. Mechanical heart valves are durable but carry increased risks of blood clotting and require life-long anticoagulation therapy, excluding them from patient subsets, such as children with critical congenital valve defects. Bioprosthetic valves are well-tolerated by the body but are not durable and exhibit accelerated calcification, particularly in younger patients (1). Hence, in the case of pediatric critical congenital valvular diseases, no ideal treatment options exist as somatic growth is required to avoid multiple reoperations as the child grows; in addition small, sizing options [ <15 mm (2)] for artificial valves remain unavailable commercially.

Regenerative medicine using tissue-engineered valvular constructs (TEVCs) may provide long lasting solutions for the treatment of critical congenital heart valve diseases in the young. TEVCs are potentially ideal because they can provide somatic growth, biological repair, and remodeling. TEVCs offer the promise of fully replacing valvular tissue functionality and the long-term integration with host tissues, without the need of surgical reinterventions.

Evidence suggests that stem cells can facilitate the acceleration of tissue production and regeneration (3). A variety of stem cells are available and used in tissue repair and regeneration including mesenchymal stem cells (MSCs). MSCs are of particular interest in cardiovascular regeneration due to their potential for self-renewal and differentiation (3). Moreover, MSCs have an added benefit of immunomodulation and angiogenesis promotion, both factors that will enhance TEVCs (3).

To develop TEVCs, it is generally accepted that mechanical forces innate to the cardiovascular system, namely, flow, stretch, and flex, can optimize *in vitro de novo* tissue growth (4–6). To facilitate this mechanical conditioning, bioreactors are commonly used to dynamically culture engineered valve tissue constructs (5, 6). The general approach to dynamically culture tissue engineered valves begins with seeding of the cells on a scaffold of choice and placing them in a bioreactor that simulates the mechanical conditions to support valvular tissue formation and phenotype of interest. Our group, and others, have previously shown that human bone marrow mesenchymal stem cells (hBM-MSCs) can produce robust engineered tissues *in vitro* (5, 7). Moreover, these seeded hBM-MSCs were able to differentiate to both endothelial cells on the surface and activated interstitial cells deeper within the constructs, similar to the native valve, when exposed to a combination of physiologically relevant cyclic flexure (1 Hz) and steady fluid-induced shear stress (4–5 dynes/cm^2^) states (5). The combination of cyclic flexure and steady flow (flex–flow) induces pulsatile and/or oscillatory flow patterns on the surfaces of TEVCs. Our work, as well as others, have shown the importance of oscillatory flow conditions on developing valve tissues (6, 8–10). However, a physiologically relevant pulsatile flow waveform may be able to induce oscillations similar to the conditions experienced in the native valve, which will mechanically condition these TEVCs. Indeed, we previously were able to show that hBM-MSCs have a significant upregulation of valve phenotypic gene expression, while valve disease-relevant genes, including osteogenic markers, were significantly downregulated after exposure to an aortic pulsatile flow profile (2D) for 48 h (11), in monolayer culture.

In the current study, we scaled our investigation to three dimensions utilizing hBM-MSC-seeded scaffolds that were mechanically conditioned using a physiologically relevant, aortic pulsatile flow waveform (11). In particular, we subsequently assessed the resulting phenotypic changes to the engineered tissue constructs after being subjected to oscillatory shear stresses resulting from the aortic flow profile, in comparison to our previous work in which oscillatory patterns in the culture media was induced under a combination of steady flow and cyclic flexure, i.e., flex–flow (cyclic flexure of 1 Hz and steady fluid-induced shear stress of 4–5 dynes/cm^2^). Notably, in the current study, the oscillations were solely fluid induced, without any structural deformation (e.g., cyclic flexure or cyclic stretch) of the specimens. He and Ku (12) have previously shown that these fluid oscillations can be quantified using the oscillatory shear index (OSI; Equation 1). Similarly, wall shear stress (WSS) has been reported to affect differentiation of valve-specific phenotypes (13). The time-averaged WSS (TAWSS) was used as metric to quantify the physiological relevance of the shear stress magnitudes on the surface of the specimens (Equation 2).

(1) $$\begin{array}{r} \text{OSI}=0.5\left(1-\frac{\left|\int_{0}^{T}\vec{\text{WSS}}\text{ dt}\right|}{\int_{0}^{T}|\vec{\text{WSS}}|\text{dt}}\right) \end{array}$$

(2) $$\begin{array}{r} \text{TAWSS}=\frac{1}{T}\underset{0}{\overset{T}{\int }}|\vec{\text{WSS}}|\text{dt} \end{array}$$

## Materials and Methods

### Computational Model Setup

**Figure 1:**
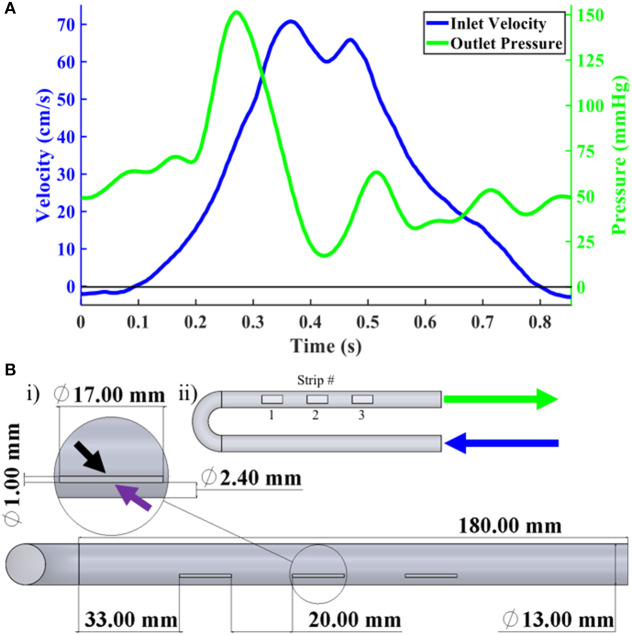
Boundary conditions and FSF Bioreactor CAD. **(A)** i) Overall CAD geometry of FSF bioreactor primarily showing the three scaffold strip locations. Magnified inset showed one of the scaffold strips with the black and purple arrows pointing to the proximal and distal sides, respectively. ii) Scaffold strip numbering designation as well as arrow labels showing inlet (blue) and outlet (green), which correspond with the information from **(B)** regarding their respective velocity and pressure profiles. **(B)** Velocity (blue), in cm/s, and pressure (green), in mmHg, waveforms recorded by our flow probe and pressure transducers.

Computational fluid dynamic (CFD) simulations were performed using commercially available software (COMSOL, Burlington, MA). An adaptation of the flow–stretch–flex (FSF) bioreactor geometry (7) was used, which primarily consisted of a U-shaped housing with a constant internal diameter of 13 mm (Figure 1A). This computational geometry had been previously used by our laboratory in numerous studies (5, 6, 9). The meshed geometry consisted of 441,000 elements and 472,000 nodes, which was based off our previous, validated mesh of the same geometry (11). Each side of the cell-seeded scaffold specimens was identified as either the proximal or distal side relative to its physical distance to the centerline axis of the bioreactor (Figure 1A).

The inlet CFD velocity waveform (Figure 1B) was derived from the experimentally captured bioreactor inlet flow waveform (see *Bioreactor Setup*); a time step of 2 ms was applied. The outlet was set to a pressure condition (Figure 1B) using an acquired pressure waveform from our transducers (Supplementary Figure A). The walls of the bioreactor were prescribed a no-slip boundary condition. The fluid was assumed to be incompressible with a constant density of 1.01 g/cm^3^, which matched that of basal cell culture media, and a dynamic viscosity of 1.27 cP (7). A convergence criterion of 1 × 10^−9^ was set for the residuals of the continuity and momentum equations. After ensuring cyclic independence between three cycles, the results from the second cycle were analyzed.

### hBM-MSCs Culture and Expansion

A total of 2 × 10^6^ hBM-MSCs (RoosterVial-hBM-1M, RoosterBio, Part No. MSC-003) were plated into six T75 flasks (*n* = 3 flasks/vial) with culture medium (h-MSC high-performance basal medium, RoosterBio, Part No. SU-005) and supplement (RoosterBooster-MSC Media Booster, RoosterBio, Part No. SU-003). The media was changed every 3 days until the hBM-MSCs were confluent, which were then harvested according to the manufacturer's protocol. In brief, the media was removed for each T75, and 3 ml of 0.25% trypsin was added and incubated in 37°C for 5 min. An equal amount of fresh media was added to the flasks and transferred to 50 ml conical tubes and centrifuged at 200 × *g* for 10 min. The supernatant was removed and resuspended in new media. The hBM-MSCs were grown to passage 6 with a total density of 18 × 10^6^ cells.

### Scaffold Preparation and Cell Seeding

An equal ratio of poly-glycolic acid (PGA) and poly-l-lactic acid (PLLA) non-woven polymer felt (PGA-PLLA; Biofelt, Biomedical Structures, Warwick, RI) (4, 14, 15), scaffolds were used for this experiment. Scaffolds were cut in rectangular-shaped strips (17 × 6 × 1 mm, *n* = 9) and gas sterilized with ethylene oxide (EtO; AN 306, Anprolene, Andersen Products Inc., HawRiver, NC) for 12 h and treated with 70% ethanol before seeding. The seeding of the hBM-MSCs on the scaffolds were similar to our previous studies (5); the supernatant was removed, and the cell pellet was resuspended with fresh Dulbecco's modified Eagle's medium (DMEM, Fisher Scientific), supplemented with 10% fetal bovine serum (Atlanta Biologics, GA, USA), 1% penicillin and streptomycin (Thermo Scientific™ HyClone™; Fisher Scientific), 2 ng/ml basic fibroblast growth factor (bFGF, Corning™; Fisher Scientific), and 82 μg/ml ascorbic acid 2 phosphate (AA2P, Sigma-Aldrich). Each scaffold was placed in a 50-ml vented conical tube (Product no. TP87050, TPP, TubeSpin Bioreactor, Zollstrasse 7, CH-8219 Trasadingen, Switzerland) and seeded with 2 × 10^6^ hBM-MSCs in 20 ml of tissue culture media. These tubes were then placed in a rotisserie (Labquake™ Rotisserie Hybridization Rotators, Thermo Scientific, USA) at 8 rpm in the incubator with controlled cell culture conditions (37°C, 5% CO_2_) for 8 days without media change.

### Bioreactor Setup

**Figure 2:**
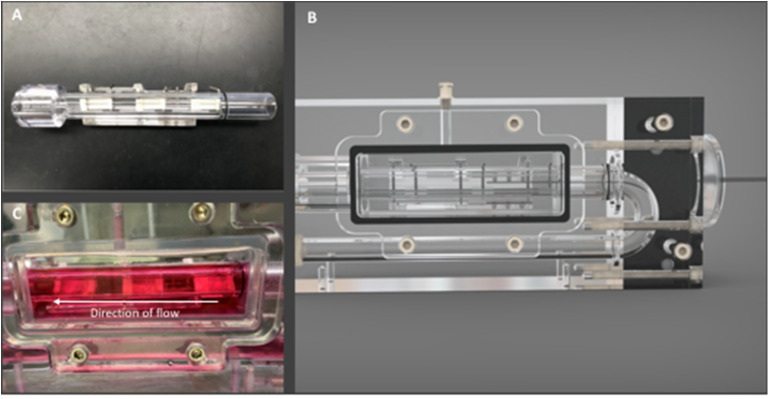
Bioreactor components. **(A)** View of scaffold holder detailing the positioning of scaffolds with metal pins, **(B)** bioreactor chamber with imaging window for direct view of scaffold holder within the chamber, and **(C)** Bioreactor imaging window with scaffolds immersed in flow media. Arrow signifies the direction of flow.

A flow perfusion bioreactor system was used to subject the seeded scaffolds to physiologically relevant pulsatile aortic flow regimens. Specific components of the system included a separable and insertable cylindrical specimen holder (Figure 2A), *U*-shaped bioreactor chambers (Figure 2B) that housed the hBM-MSC-seeded scaffolds (7), and flow that was facilitated by a programmable pulsatile pump system (ViVitro Labs Inc., Victoria, BC, Canada). Three scaffolds were placed in each bioreactor chamber in a parallel configuration to the flow direction and were fully immersed in the media solution (Figure 2C). Note that this bioreactor system and its applicability for heart valve tissue engineering purposes has been previously validated (7).

**Figure 3:**
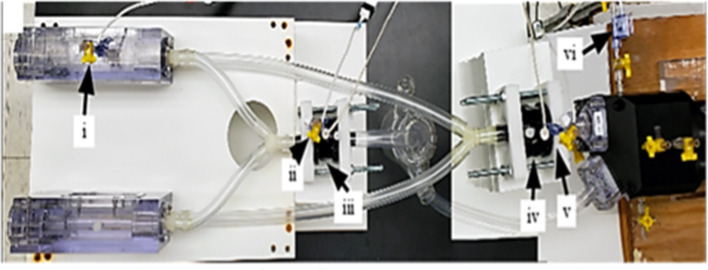
Assembled bioreactor pulsatile flow loop. The major components of the bioreactor include: the **(i)** pressure sensor at bioreactor chamber, **(ii)** pressure sensor at bioreactor exit, **(iii)** flow probe at bioreactor exit, **(iv)** flow probe at pump exit, **(v)** pressure sensor at pump exit, and the **(vi)** pressure sensor inserted into pump-head interior.

**Figure 4:**
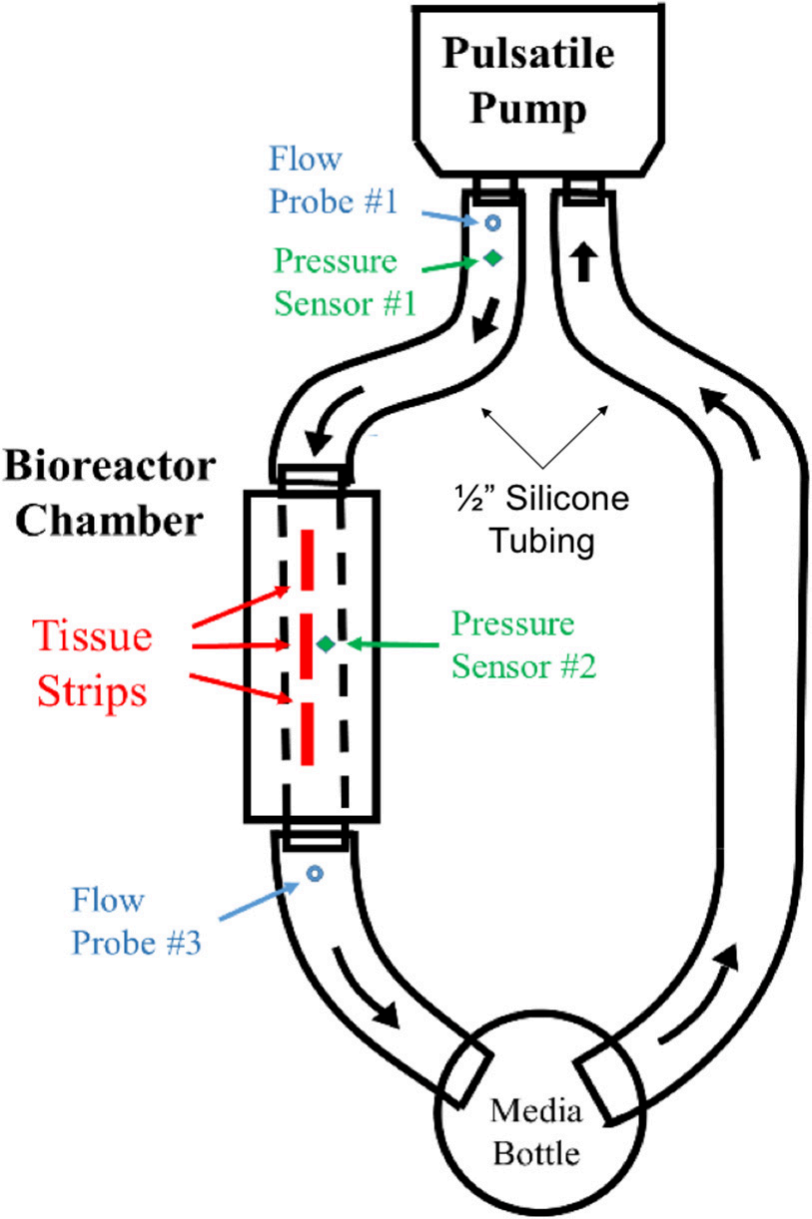
Flow diagram for FSF bioreactor dynamic conditioning system. Pressure and flow sensors at pump outlet (V1, P1) and at a pressure sensor at bioreactor chamber (P2) were used to validate flow velocities within the bioreactor chamber.

The long axis of each scaffold specimen was oriented parallel to the direction of flow, with flow passing over their surfaces. The pump was connected via silicone tubing (Masterflex 96410-35, Cole-Parmer, Vernon Hills, IL) to two bioreactor chambers in a parallel configuration (Figure 3). The diameter of the tubing was 0.5 in, reflecting a spatial dimension closer to that of the descending aorta [normal diameter of the aorta ranges between 0.3″ and 0.6″ from infant to adolescent (16)]. Other components of the flow loop (Figure 4) included a glass media bottle, flow probes (Carolina Medical Electronics Inc., NC, USA), pressure sensors, and test software (Vivitest, ViVitro Systems), used to obtain the relevant hydrodynamic data during the flow and pressure testing phase.

To validate that the required flow waveform imparted by the pulsatile pump (S35-HR70-SV40) physiological waveform (Vivitest software, Vivtro Laboratories) was reproduced within the bioreactor chamber at the site of the hBM-MSC-seeded scaffolds, flow and pressure sensors were placed at the pump exit location to record pump output flowrate (*Q*_1_) and pressure (*P*_1_) data (Figures 3iv,v). Note also that for the purposes of validation, only a single chamber (as opposed to two parallel chambers used in the tissue engineering experiments; Figure 3) was used. The flow velocity (*V*_1_) at the pump output location was subsequently computed using conservation of mass principles as:

(3) $$\begin{array}{r} Q_{1}=V_{1}A_{1} \end{array}$$

A pressure sensor was inserted in the bioreactor housing at the mid-point directly overhead of the scaffold flow field (*P*_2_ location; Figure 3i). With the application of the Bernoulli's equation (Equation 4), the velocity within the bioreactor chamber (*V*_2_) was estimated (Equation 5) and its corresponding flow rate subsequently computed (*Q*_2_; Equation 6). Additionally, a flow probe was placed at the exit of the bioreactor chamber (Figure 3ii) to further validate flow conditions (*Q*_3_) within the chamber and scaffold specimens.

(4) $$\begin{array}{r} P_{1}+\frac{\rho V_{1}^{2}}{2}+\rho gh_{1}=P_{2}+\frac{\rho V_{2}^{2}}{2}+\rho gh_{2} \end{array}$$

(5) $$\begin{array}{r} V_{2}=\sqrt{V_{1}^{2}+\frac{2}{\rho }\left(P_{1}-P_{2}\right)} \end{array}$$

(6) $$\begin{array}{r} \;Q_{2}=V_{2}A_{2} \end{array}$$

Flow and pressure data for 10 pulse cycles were collected and averaged to produce flowrate waveforms for pump exit (*V*_1_ location; Figure 3iv), bioreactor chamber (V_2_ location; Figure 3i) and bioreactor exit (*V*_3_ location; Figure 3iii). These three flow profiles were then plotted to compare the pump output waveform to the corresponding pulsatile flow field that was presented at the site of the hBM-MSC-seeded scaffold specimens and at the exit location of the bioreactor chamber (Figure 3). A percentage error in maximum and mean flowrates between the pump output region and the two secondary locations were subsequently computed as:

(7) $$\begin{array}{r} \text{Max Flow Rate \% Error:}\\ \;\frac{\text{max flow @ pump exit}-\text{max flow @ specimen}}{\text{max flow @ pump exit}}\;\times \;100\% \end{array}$$

(8) $$\begin{array}{r} \text{Mean Flow Rate \% Error:}\\ \;\frac{\text{mean flow @ pump exit}-\text{mean flow @ specimen}}{\text{mean flow @ pump exit}}\;\times \;100\% \end{array}$$

### Tissue Engineering Experiments

After the hBM-MSCs were seeded on the scaffolds, the specimens were subjected to 8 days of rotisserie culture; next, they were split into two groups: (1) no flow (static controls, *n* = 3) and (2) physiological oscillatory flow (bioreactor group, *n* = 3/chamber). The cell-seeded specimens in the no flow group (hereby referred to as the “static” group) were immersed in media within a 6-well plate and left within in a tissue culture incubator for an additional 14 days without media change. Meanwhile for the oscillatory flow group, the cell-seeded specimens were sutured on both ends to metallic springs and inserted onto stationary rods within a *U*-shaped bioreactor device (drawings and details in (7)). The bioreactors were then connected to a pneumatic piston pulsatile flow pump, with a pump head module (ViVitro Labs Inc., Victoria, BC, Canada) that housed the tubing to transport media to the specimens housed within the bioreactors (one bioreactor chamber contained another scaffold material that was conditioned identically, but not connected to the present study). Physiologically relevant fluid-induced wall shear stresses (WSS) of 3–9 dynes/cm^2^ have been shown to be conducive to proper cell conditioning (17). Similarly, fluid oscillations, measured with oscillatory shear index (OSI), between 0.18 and 0.23 have been reported to assist in promoting genes that support the valve phenotype (11). Hence, an aortic flow waveform (11), which could elicit valve relevant of TAWSS and OSI (as determined from the CFD simulations; *Computational Model Setup*) was applied to the bioreactor for an additional 14 days, beyond the initial 8-days rotisserie culture period. Total culture time of the engineered tissue constructs in both the static and bioreactor time was therefore 22 days (using the same media formulation as in *Scaffold Preparation and Cell Seeding*), which consisted of 8 days rotisserie culture followed by either an additional 14 days static or 14 days pulsatile flow culture. Viability of cells after bioreactor culture was verified independently via observation of cell proliferation prior and after a 14-days oscillatory flow conditioning experiment with stem cells (unpublished observation).

### Immunofluorescence

Once the experiment was complete, the scaffolds were prepared for immunofluorescence assessment similar to our previous work (18). In brief, the scaffolds were fixed in formalin (10% *v*/*v*) for 24 h followed by embedding in a slow freeze process in optimal cutting temperature (OCT). The embedded samples were then placed in −80°C overnight and then sliced (16 μm of thickness) and placed on glass slides (TruBond 380, Newcomer Supply, Middleton, “Wisconsin”) and left to dry at room temperature to allow for immunofluorescence staining. The slides were stained with primary antibodies for valvular components, including endothelial and interstitial cells; CD31 was used as an endothelial cell marker (Invitrogen, REF: PA5-14372), and alpha smooth muscle actin (α-SMA) was used as an interstitial cell marker (Invitrogen, REF: 14-9760-82), separately. Donkey antirabbit (Abcam, ab150073) and donkey antimouse (Abcam, ab150108) were used as secondary antibodies, respectively, for CD31 and α-SMA. Immunofluorescence images were captured with confocal microscopy (Nikon Eclipse T*i*, Minato, Tokyo, Japan).

All samples from the static control and oscillatory flow groups were stained for CD 31 and α-SMA immunostaining at the same time. On the other hand, in order to make observations with previously reported (5) samples from flex–flow experiments and positive porcine valve (PHV) controls, the signal intensities in all groups were normalized relative to the average intensity of the static samples across the previous and the present investigations.

### Image Analysis

**Figure 5:**
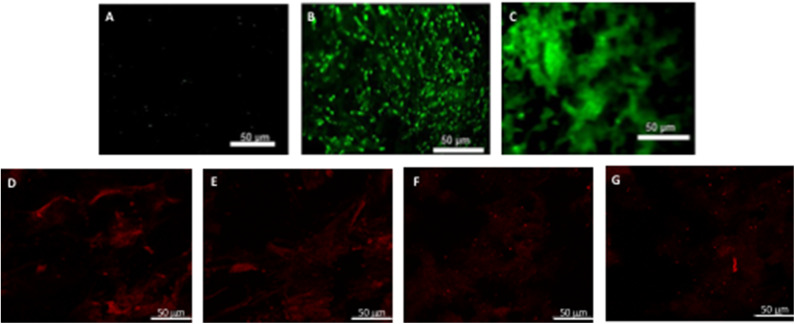
hBM-MSCs differentiation toward α-SMA phenotype. PGA-PLLA scaffolds seeded with hBM-MSCs (5) demonstrated the presence of α-SMA phenotype in **(A)** static, **(B)** flex-flow and **(C)** PHV groups; the same cell-seeded scaffolds from our experiments focusing on **(D,E)** static, and **(F,G)** physiologically-relevant oscillatory flow groups demonstrate the presence of α-SMA phenotype. Image magnification of 72X.

**Figure 6:**
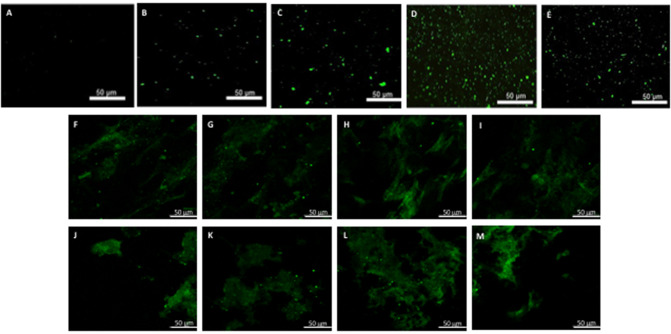
hBM-MSCs differentiation toward CD31 phenotype. PGA-PLLA scaffolds seeded with hBM-MSCs (5) demonstrated the presence of CD31 phenotype in **(A)** static, **(B,C)** flex-flow, and **(D,E)** PHV groups; the same cell-seeded scaffolds from our experiments focusing on **(F–I)** static and **(J–M)** physiologically-relevant oscillatory flow groups demonstrate the presence of CD31 phenotype. Image magnification of 72X.

Images from previous work in our laboratory were gathered for comparison purposes (static, flex–flow, and porcine native heart valve) [Figures 5A–C, 6A–E, (5)], while confocal images of α-SMA and CD31 were taken on our experimental groups of static (no flow) and bioreactor (oscillatory flow) groups (Figures 5D–G, 6F–M). All images were sized to a resolution of 1,024 × 690 pixels to be able to make these comparisons. The static images, from both our experiments and from our previous work, were merged together into and averaged static image (MATLAB, Mathworks, Natick, MA). The mean intensity of this image was determined and was used to normalize the average static image. This image was used, with its mean intensity, to normalize all the other groups of interest (i.e., oscillatory flow, flex–flow, and PHV). Heat maps were computed (MATLAB) to demonstrate the intensity differences in the pixels of each image, maintaining the same scale throughout. This procedure was used for α-SMA and CD31 images independently.

### Protein Quantification

**Figure 7:**
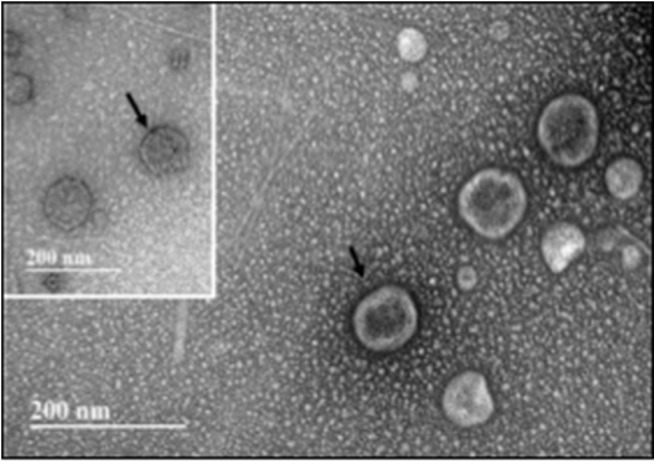
Transmission electron microscopy (TEM) of exosomes isolated from hBM-MSCs after an initial 8 days of rotisserie culture and followed by oscillatory flow conditioning in our bioreactor for 2 weeks. Insert: Immunogold labeling with CD 9 antibody. Arrow indicates site of CD9-Immunogold labeling. Scale bar = 200 nm.

Conditioned media was collected from static, 8-days rotisserie, and oscillatory flow groups and centrifuged at 3,000 × *g* for 15 min to remove cell debris. An appropriate volume of ExoQuick-TC solution (System Biosciences, Palo Alto, CA) was added to each group to precipitate the exosomes that were secreted by hBM-MSCs. The media/ExoQuick-TC mixture was refrigerated overnight (~16 h) at 4°C and then centrifuged at 1,500 × *g* for 30 min. The supernatant was removed, and the residual of ExoQuick-TC precipitation solution was aspirated after the centrifugation at 1,500 × *g* for 5 min. Exosome pellets were resuspended in 250 μl phosphate-buffered saline (PBS) and filtered with 0.2-μm syringe filters (Thermo Scientific™ Nalgene™, Fisher Scientific, Hampton, NH) (Figure 7). To extract proteins from the membrane of exosomes, radioimmunoprecipitation assay (RIPA) buffer (Fisher Scientific) was added at the ratio 1:1. The mixture was gently shaken on ice for 15 min and centrifuged at 14,000 × *g* for 15 min. DC Protein Assay (Bio-Rad, Hercules, CA, USA) was used to determine protein concentration. The absorbance of the samples was read at 800 nm wavelength by a spectrophotometer (Synergy HT, BioTek Instruments Inc., Winooski, VT).

### Liquid Chromatography–Tandem Mass Spectrometry Analysis

Samples were digested according to the protocol of In-Solution Tryptic Digestion and Guanidination Kit (Thermo Scientific™, Fisher Scientific). In brief, samples were first reduced and denatured with 15 μl digestion buffer and 1.5 μl reducing buffer and incubated at 95°C for 5 min. Afterwards, 3 μl alkylation buffer was added to the samples to perform the process of alkylation in the dark at room temperature for 20 min. Second, 1 μl activated trypsin was added for digestion and incubated at 37°C for 3 min. Additional 1 μl activated trypsin was added, and the samples were incubated overnight (~18 h) at 30°C. Lastly, 3 μl formic acid (Thermo Scientific™ Pierce™ LC-MS Grade, Fisher Scientific) was added to stop the reaction. Liquid chromatography–tandem mass spectrometry (LC-MSMS) analysis was conducted on a Bruker tims-TOF UHR MS (Bruker Scientific. Billerica, MA) instrument operated in positive (+) ion mode in the mass range from 300 to 2,400. Chromatography separation was conducted utilizing a 46-min-long LC method with optima grade water (0.1% formic acid) as the aqueous phase and optima grade acetonitrile with (0.1% formic) as the organic phase on a Shimadzu Prominence HPLC (Tokyo, Japan) equipped with a Waters XBridge C18 Proteomics Column (Milford, MA). Moreover, the in-solution digested extracts were diluted 1:10 in 50:50 MeOH/water [0.1% formic acid (FA)] and stored in silanized glass inserts placed inside a sampling vial, which were then loaded onto a high-performance liquid chromatography (HPLC) autosampler (Shimadzu Prominence, Nakagyo-ku, Japan). Thereafter, a 20-μl aliquot was injected into the HPLC for separation prior to mass spectrometry (MS) analysis. The MS was calibrated with a tuning mix solution (Agilent, Santa Clara, CA) with a reported standard deviation <1 ppm. Tandem MS/MS peptide fragmentation was conducted with collision-induced dissociation of the 25 most abundant precursors. After data acquisition, the obtained raw mass spectrometry data were processed using Peaks Studio 8.5 (Bioinformatics Solution Inc., Waterloo, ON, Canada) for protein identification, peptide sequencing, and label-free quantification.

### Statistical Analysis

All results were reported as means ± standard error of mean. The average intensities of the images (averages static, oscillatory flow) were statistically analyzed via a *t*-test whenever normality was held; otherwise, a non-parametric test was used (Minitab, Inc., State College, PA). For fibronectin protein intensity from the collected media a log-transformed dataset was used to perform a parametric ANOVA and Tukey's honestly significant difference (HSD). Statistical significance between any two given groups was found to have occurred when *p* < 0.05). Note that comparisons to our previously published work on analogous flex–flow experiments (5) was done observationally (rather than statistically) due to a limited ability to increase sample size from a work that was published 5 years ago.

## Results

### Velocity

**Figure 8:**
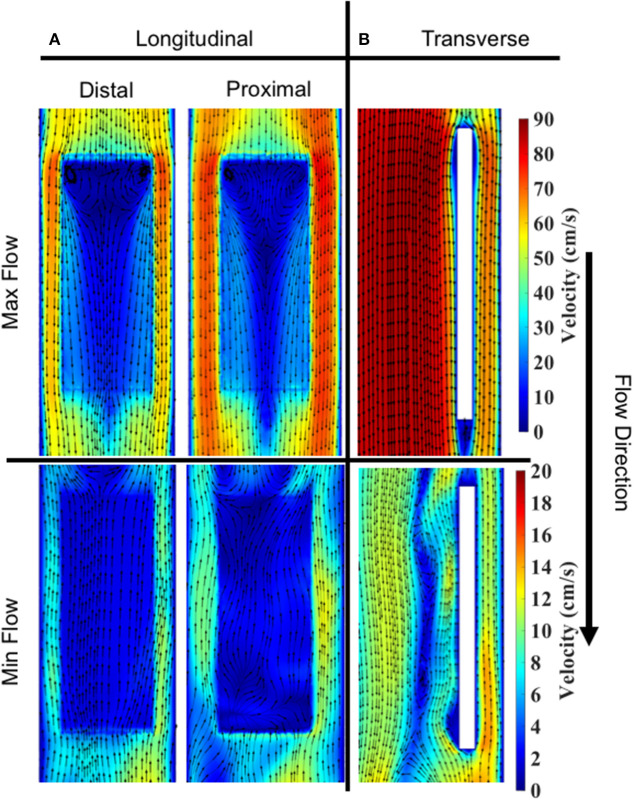
Velocity contours and streamlines on specimen #2. Max and min flow corresponded to ~0.368 and ~0.83 s, respectively, in the enforced velocity waveform. **(A)** Longitudinal slice showing a comparison of streamlines on distal vs. proximal surfaces. Cut planes were taken 200 μm above and below the specimen to extract velocity contours. **(B)** Transverse slice showing a comparison of streamlines during Max vs. Min flow. Cut planes were taken through the center of specimen.

Velocity contours with streamlines are presented in Figure 8 during two time points of the flow waveform (maximum and minimum flow; Figure 1B). Two views are shown—a longitudinal (Figure 8) and transverse view (Figure 8). Overall, the flow streamlines exhibited uniformity in their pattern around the specimen surfaces. At the instance of maximum flow, in both the distal and proximal sides, there were areas of local flow reversal at the initial plane of fluid contact with each sample. On the other hand, at the instance of minimum flow, the flow was largely reversed on the surface of the specimens.

### Specimen Shear Stress

**Figure 9:**
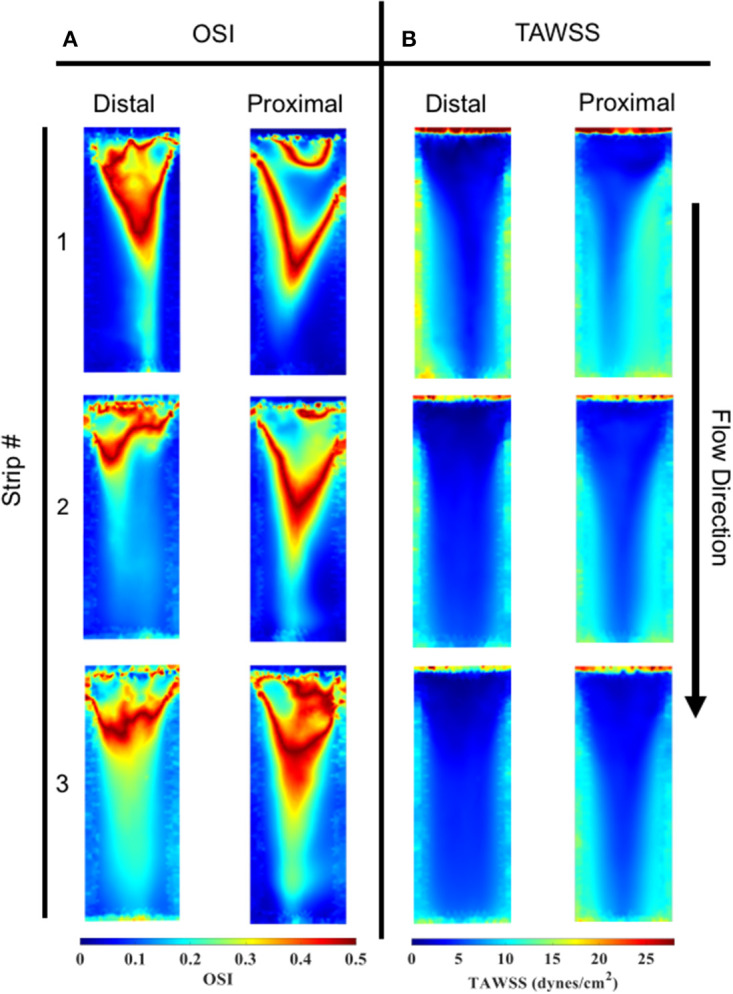
OSI and TAWSS contours across all three specimens for both the distal and proximal sides. **(A)** In order of specimen number OSI averages were; Distal: 0.145, 0.170, and 0.211, Proximal: 0.148, 0.163, and 0.189. **(B)** In order of specimen number TAWSS (dynes/cm^2^) averages were: Distal: 8.985, 7.703, and 6.122, Proximal: 9.057, 7.944, and 7.549.

Spatial distributions of TAWSS for both the distal and proximal walls of the bioreactor scaffolds are shown (Figure 9B). Overall, the proximal side had a slightly larger TAWSS (mean ± SEM) than the distal side, 8.18 ± 0.26 vs. 7.60 ± 0.48 dynes/cm^2^ (*n* = 3). However, the first specimen on both the distal and proximal sides had a larger TAWSS than the proceeding two cell-seeded scaffolds.

### Oscillatory Shear Index

OSI distributions are shown (Figure 9A) for both the distal and proximal walls of the bioreactor scaffold strips. Overall, the proximal and distal side showed comparable OSI (mean ± SEM), 0.17 ± 0.007 vs. 0.18 ± 0.01. Upstream regions of all three specimens had a higher OSI compared to its corresponding downstream location. There was also the presence of a “V”-shaped OSI contour present on both distal and proximal sides of the specimens; a similar “V” shape was exhibited when looking at the same location's TAWSS (Figure 9B).

### Bioreactor Setup

**Figure 10:**
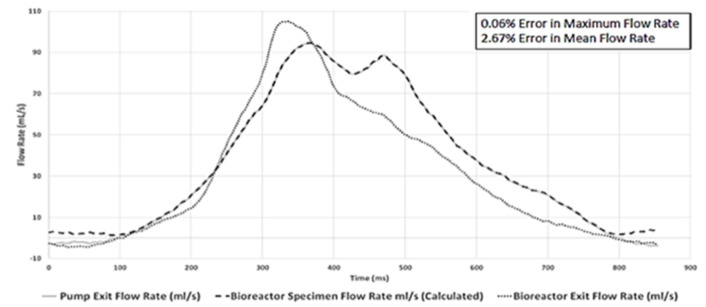
Physiologically-relevant pulsatile flow profile comparison at three flow-loop locations. Close agreements between the pump exit and the bioreactor specimen location flow rates (peak flow error 0.06%; mean flow error 2.67%) were found. This demonstrated that the bioreactor chamber-housed, cell-seeded scaffold specimens would receive the intended aortic pulsatile flow waveform utilized in the tissue engineering experiments.

Waveform prediction (at the vicinity of the housed bioreactor samples; Figures 3, 4) and measurements (at the pump and bioreactor exits; Figure 3) for the cardiac cycle showed close agreement with one another (Figure 10, Supplementary Figure A). Quantitative analysis of this flow data showed a 0.06% error in maximum flowrate and a 2.67% error in mean flowrates between the pump output and the temporal flowrates at the specimen locations, indicating that the cell-seeded constructs would receive the intended aortic pulsatile flow profile during the bioreactor experiments. Thus, overall, the pulsatile bioreactor system was able to recreate the flow profiles imparted by the pump within the bioreactor chamber with a high degree of fidelity.

### Immunofluorescence and Image Analysis

**Figure 11:**
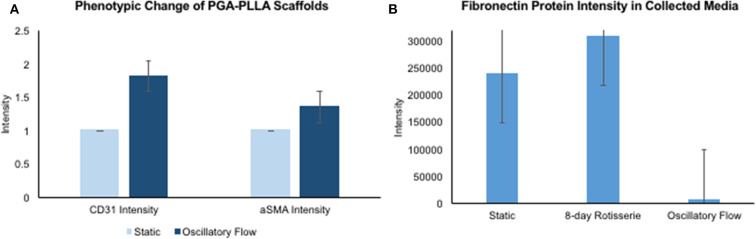
hBM-MSCs differentiation toward α-SMA and CD31 valvular phenotypes and fibronectin protein intensity in collected media. PGA-PLLA scaffolds seeded with hBM-MSCs demonstrating **(A)** average intensity values of α-SMA and CD31 in static and oscillatory flow groups. There was a significant difference (*p* < 0.05) in both α-SMA and CD31 phenotypes intensities between the groups. In the oscillatory flow group there was an increase in both α-SMA and CD31 compared to the static group (significant, *p* < 0.05). **(B)** Media was collected and fibronectin intensities were assessed for static, 8-day rotisserie and oscillatory flow groups. The oscillatory flow group was significantly lower (*p* < 0.05) than both the static and 8-day rotisserie groups. *P*-values and boxplots for specific statistical analysis can be seen in Supplementary Figures B–D.

**Figure 12:**
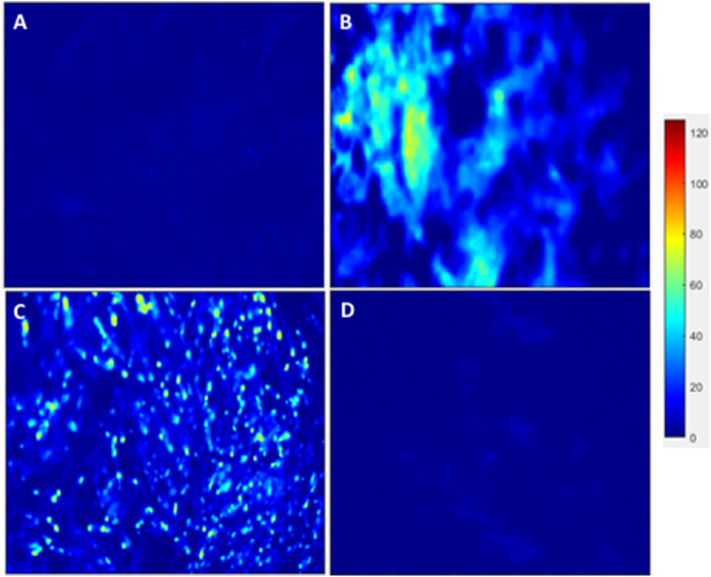
hBM-MSCs differentiation toward α-SMA heat map. PGA-PLLA scaffolds seeded with hBM-MSCs from previous experiments (5) compared to our experiments demonstrating heat maps of α-SMA in averaged **(A)** static, **(B)** PHV, **(C)** flex-flow, and **(D)** oscillatory flow groups. Resolution of 1,024 × 690 pixels.

**Figure 13:**
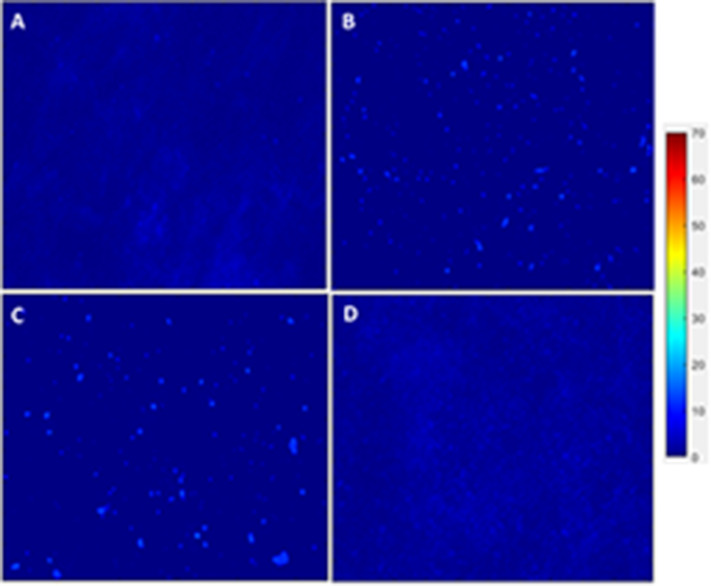
hBM-MSCs differentiation toward CD31 heat map. PGA-PLLA scaffolds seeded with hBM-MSCs from previous experiments (5) compared to our experiments demonstrating heat maps of CD31 in averaged **(A)** static, **(B)** PHV, **(C)** flex-flow, and **(D)** oscillatory flow groups. Resolution of 1,024 × 690 pixels.

After analyzing the static, flex–flow, and PHV images captured from the previous studies (Figures 5, 6) conducted in our laboratory (5), the average intensities were calculated for α-SMA and CD31 (for our groups, i.e., static and oscillatory flow) (Figure 11A), and heat maps based off of the florescence signal intensities were computed (MATLAB) for all groups (i.e., static, PHV, flex–flow, and oscillatory flow) (Figures 12, 13). Observationally, the flex–flow group (from previous studies) had a larger α-SMA intensity than our oscillatory flow group (Figure 12). For statistical comparisons, only our static and oscillatory flow groups were assessed due to sample size limitations (static, *n* = 12; oscillatory flow, *n* = 10 or 16; flex–flow, *n* = 1; PHV, *n* = 1). For α-SMA, the average intensities were found to be 1.0 ± 0.15 and 1.4 ± 0.11 for static and oscillatory flow, respectively (Figure 11A). Similarly, the average intensities were only computed for static and oscillatory flow groups since both the flex–flow and PHV groups had small samples (*n* = 2). It was found that the average intensities were 1.0 ± 0.18 and 1.8 ± 0.26 for static (*n* = 12) and oscillatory flow (*n* = 10), respectively (Figure 11A). The *p*-values are given in Supplementary Figures B, C. The corresponding heat maps for both α-SMA and CD31 are shown in Figures 12, 13.

### Protein Quantification

Once the media was collected form all three groups, i.e., static, 8-days rotisserie, and oscillatory flow, the protein intensity was analyzed through mass spectrometry. One protein of interest was fibronectin, which demonstrated a significantly lower (*p* < 0.05) intensity of fibronectin in the oscillatory flow group compared to the static and 8-days rotisserie groups (Figure 11B). There was no significance (*p* > 0.05) between the static and 8-days rotisserie group (Figure 11B). The *p*-values and further details on the statistical analysis are given in Supplementary Figure D.

## Discussion

Congenital heart valve defects in the young have no viable treatment options currently available mainly due to limitations in current artificial valve sizing options and their inability to support somatic growth. Regenerative medicine using TEVCs may provide long-lasting solutions since TEVCs can facilitate provisions for somatic growth, biological repair, and remodeling. MSCs are one of the most promising and suitable cell types for regeneration since they can differentiate into endothelial and interstitial-line cell subtypes, which reside in heart valves. The microenvironment of these cells includes both biochemical factors and hemodynamic forces that influence differentiation. On the other hand, a standard reproducible protocol to develop a valvular construct to be used *in vivo* for longitudinal purpose is lacking (19). The objective of the current study was to determine whether using a physiologically relevant pulsatile flow profile that would elicit oscillatory shear stresses on MSC-seeded scaffold constructs could advance a tissue-engineered valve construct's phenotype within the *in vitro* domain prior to implantation. Phenotypic matching of the implant with host tissues is an important attribute in subsequent *de novo* tissue remodeling *in vivo*, due to chemotactic events that are initiated by the implanted engineered extracellular matrix and cells, which may accelerate host tissue regeneration due to its niche (20). Such an outcome is especially critical in situations such as heart valve replacement in children, where support of somatic growth by the engineered valve construct is critically needed.

Our immunofluorescence intensity assessment demonstrated that the α-SMA quantification between the groups (i.e., average static and oscillatory flow) were significantly different (*p* < 0.05) (Figure 11A and Supplementary Figure B); similarly, the CD31 levels were found to be significantly different (*p* < 0.05) (Figure 11A and Supplementary Figure C). These findings can be attributed to the microenvironment that the hBM-MSCs were exposed to. The physiological nature of the aortic oscillatory flow waveform that was used during our experiment was intended to mimic hemodynamic shearing forces, which occur physiologically *in vivo*. Shear stresses that the hBM-MSCs are exposed to play a critical role in differentiating the cells toward the endothelial phenotype (19, 21–23). This supports our finding that the oscillatory flow group had a higher CD31 expression (endothelial phenotype). Studies in valve tissue engineering have suggested that valvular endothelial cells (VECs; CD31 marker) are the critical factor needed when compared to the valvular interstitial cells (VICs; α-SMA marker) for *in vivo* implantation (24–27). In one study, autologous, ovine endothelial cell-seeded scaffolds were exposed to pulsatile flow conditioning for a week and demonstrated a complete endothelial layer on the leaflet surface but no presence of interstitial cells (25, 27). The conditioned valves were implanted in the descending aorta of a sheep for 3 months, functioning normally, with an observed endothelial layer but minimal leaflet interstitial recellularization (25, 27). Yet, another study investigated mechanically conditioned (rotisserie culture for 24 h followed by continuous flow at 15 ml/min with the circulation maintained for 21 days) tissue-engineered valves in two pediatric patients using a decellularized human pulmonary valve scaffold seeded with autologous mononuclear cells isolated from peripheral blood (24, 27). These scaffolds were monolayer with its surface positive for EC phenotypes, yet exhibited a complete absence of interstitial cells (24, 27). After a 3.5-years follow-up, both patients had normal valve function without any complications (24, 27). Both these studies suggest that with solely the presence of the endothelial phenotype, a valve construct can be implanted and function normally; nevertheless, the efficacy of these valves longer term still remains unclear. In the context of our study, we have demonstrated for the first time that physiologically relevant oscillatory shear stresses alone (overall mean, time-averaged shear stress, ~7.9 dynes/cm^2^; overall mean, oscillatory shear index, ~0.18), with the absence of solid stresses in the mechanical conditioning protocol can augment the endothelial phenotype while restricting the activated smooth muscle phenotype (α-SMA) to relatively low levels. Note that oscillatory flow is distinct from pulsatile flow in that the latter may not necessarily induce oscillations in the fluid.

Furthermore, it has been reported that while shear stress promotes endothelial differentiation (CD31), it downregulates the differentiation toward smooth muscle cell phenotype (α-SMA) (Figures 12, 13) (19, 28, 29). While the flex–flow group had greater α-SMA expression compared to our oscillatory flow group (Figure 12), this may not necessarily be desirable since α-SMA is both an indicator of healthy tissue remodeling as well as disease (27). Moreover, the extent of α-SMA necessary during engineered valve tissue construct to promote healthy (as opposed to pathological) remodeling after implantation is at present unknown. Finally, we also found that our oscillatory flow group had a significantly lower (*p* < 0.05) fibronectin expression intensity compared to our static and 8-days rotisserie groups (Figure 11B), which may be a sign of a healthy tissue remodeling, since fibronectin has been linked to transforming growth factor-β1 (TGF-β1) binding interactions (30); in turn, mechanically stimulated TGF-β1 signaling is associated with valve calcification (31).

Our study had the following limitations: The culture media used in our bioreactor system was void of two ingredients (AA2P and bFGF) compared to our previous work (5). However, these biochemical agents are primarily to augment collagen content, which was not the focus of the current study (14). Moreover, immunostaining intensities of engineered valve constructs in the present investigation were compared to corresponding intensities in our previous work on flex–flow and PHV groups (5) observationally (as opposed to statistically), as we were limited by the number of images (and hence sample size) available in the latter.

In conclusion, in our previous work, we found that the samples from the flex–flow group exhibited a valve-like distribution of cells that expressed endothelial (CD31) and myofibroblast (α-SMA) phenotypes within the surface and interstitial layers, respectively (5). We interpret that this was likely due to the presence of oscillatory shear stresses (overall mean, OSI ~0.11) on the sample, induced during the time-varying flow on the sample surface, which was created via the cyclic flexure of the immersed samples in the steady flow environment (5). In the current study, we similarly found that physiologically relevant, flow oscillations (overall, mean OSI ~0.18) that were directly created via the aortic flow waveform augmented the CD31 phenotype. In contrast to flex–flow conditions, however, oscillatory flow without the presence of concomitant flexural stresses served to substantially reduce the α-SMA phenotype. Therefore, physiologically relevant oscillatory flow alone may serve as a means to promote controlled *in vitro* valve tissue regeneration, by enhancing the endothelial phenotype while restricting myofibroblast phenotypic expression. Since α-SMA is indicative of both normal as well as pathological tissue remodeling activity, it would therefore be more prudent to minimize α-SMA expression while augmenting other valvular parameters that can be achieved via mechanical conditioning. However, since solid stresses may serve to also considerably increase α-SMA, a compromise can be achieved via the sole application of physiologically relevant oscillatory fluid-induced stresses, without structural deformation of the specimens. Lastly, a very low level (relative to controls) of fibronectin expression found within the conditioned media of the oscillatory flow-stimulated, hBM-MSC-seeded scaffolds is suggestive of the potential for physiologically relevant oscillatory shear stresses to minimize the risk of provoking TGF-β-mediated, pathological valve remodeling activity following *in vivo* translation.

## Data Availability Statement

The datasets in this study are available by request to the corresponding author.

## Author Contributions

BG carried out the static and bioreactor experiments, immunostaining, performed confocal microscopy, wrote parts of the manuscript, including the primary preparation of references, and figures as well as manuscript organization. MP-N set-up the bioreactor system to conduct flow and pressure measurements, performed the subsequent data analyses to verify the accuracy of the flow field within the bioreactor system, and wrote parts of the manuscript. AM performed all the CFD-related set-up, simulations and post-processing, assisted in flow and pressure testing, and wrote parts of the manuscript. MP was responsible for generating the heat maps from the florescence signal intensities derived from the α-SMA and CD31 images. Y-ML performed protein quantification and mass spectrometry on collected media samples and wrote parts of the manuscript. C-PH assisted in the bioreactor experiments, operation of the pulsatile flow pump as well as in the acquisition of raw data during flow, and pressure testing. AR and AC assisted in isolation of exosomes from the conditioned media and with the process of protein quantification, as well as sample preparation for mass spectrometry. MG and FF-L performed mass spectrometry analysis and wrote parts of the manuscript. FG identified the appropriate statistical methods and conducted the statistical analyses for the study. SR conceived the study, wrote parts of the manuscript, reviewed all procedures, and verified the writing for factual accuracy.

## Conflict of Interest

The authors declare that the research was conducted in the absence of any commercial or financial relationships that could be construed as a potential conflict of interest.
