# Supplementary figures and images for: Physiologically Relevant Fluid-Induced Oscillatory Shear Stress Stimulation of Mesenchymal Stem Cells Enhances the Engineered Valve Matrix Phenotype
Source: Front Cardiovasc Med. 2020 May 19;7:69. doi: 10.3389/fcvm.2020.00069 (PMC7248568; doi:10.3389/fcvm.2020.00069)

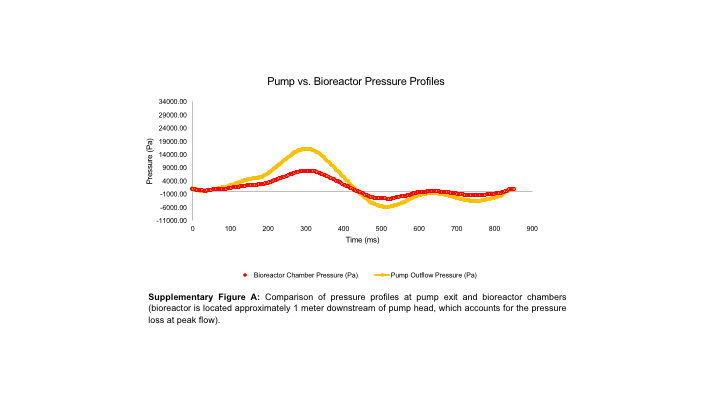

Supplement: Supplementary file 1 [file Image_1.TIFF]

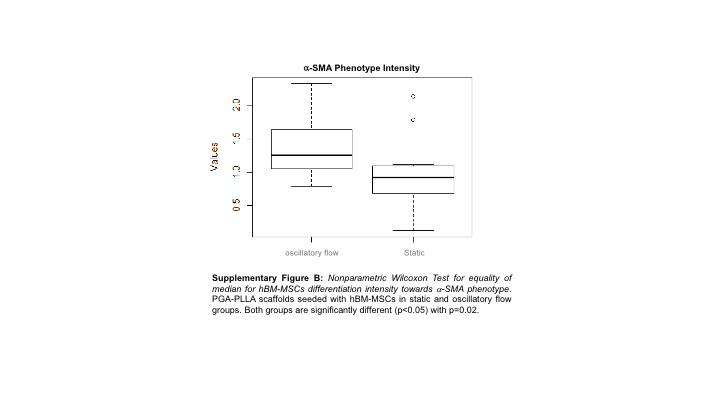

Supplement: Supplementary file 2 [file Image_2.TIFF]

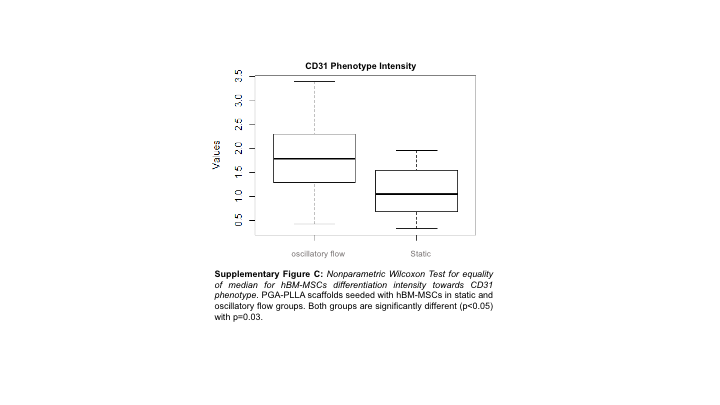

Supplement: Supplementary file 3 [file Image_3.TIFF]

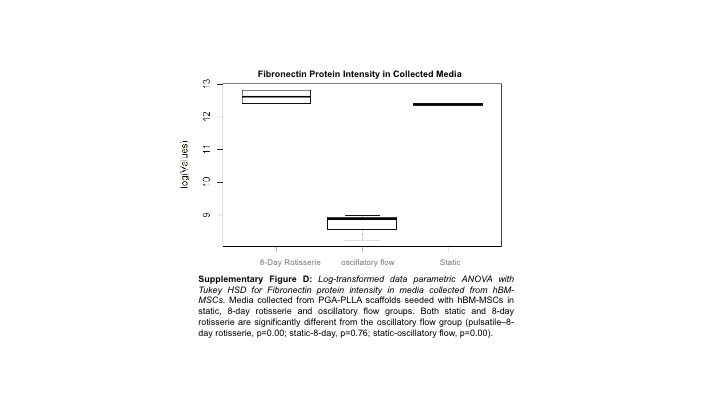

Supplement: Supplementary file 4 [file Image_4.TIFF]
